# Segmentation of Uterus Using Laparoscopic Ultrasound by an Image-Based Active Contour Approach for Guiding Gynecological Diagnosis and Surgery

**DOI:** 10.1371/journal.pone.0141046

**Published:** 2015-10-30

**Authors:** Xue-Hao Gong, Jun Lu, Jin Liu, Ying-Yuan Deng, Wei-Zong Liu, Xian Huang, Yong-Heng Yang, Qin Xu, Zhi-Ying Yu

**Affiliations:** 1 Department of Ultrasound, First Affiliated Hospital of Shenzhen University, Second People’s Hospital of Shenzhen, Shenzhen, China; 2 Department of Ultrasound, Second Clinical College of Jinan University, People’s Hospital of Shenzhen, Shenzhen, China; 3 Department of Obstetrics & Gynecology, First Affiliated Hospital of Shenzhen University, Shenzhen, China; Shenzhen institutes of advanced technology, CHINA

## Abstract

In laparoscopic gynecologic surgery, ultrasound has been typically implemented to diagnose urological and gynecological conditions. We applied laparoscopic ultrasonography (using Esaote 7.5~10MHz laparoscopic transducer) on the retrospective analyses of 42 women subjects during laparoscopic extirpation and excision of gynecological tumors in our hospital from August 2011 to August 2013. The objective of our research is to develop robust segmentation technique for isolation and identification of the uterus from the ultrasound images, so as to assess, locate and guide in removing the lesions during laparoscopic operations. Our method enables segmentation of the uterus by the active contour algorithm. We evaluated 42 in-vivo laparoscopic images acquired from the 42 patients (age 39.1 ± 7.2 years old) and selected images pertaining to 4 cases of congenital uterine malformations and 2 cases of pelvic adhesions masses. These cases (*n* = 6) were used for our uterus segmentation experiments. Based on them, the active contour method was compared with the manual segmentation method by a medical expert using linear regression and the Bland-Altman analysis (used to measure the correlation and the agreement). Then, the Dice and Jaccard indices are computed for measuring the similarity of uterus segmented between computational and manual methods. Good correlation was achieved whereby 84%–92% results fall within the 95% confidence interval in the Student t-test) and we demonstrate that the proposed segmentation method of uterus using laparoscopic images is effective.

## Introduction

The technological improvements of the endoscopic transducer has facilitated the application of B-scan laparoscopic ultrasound as a preoperative and intra operative device for supporting surgical management, as well as control and assessment. In the field of gynecologic surgery, it can be used to facilitate gynecologists to find small residual lesions under laparoscopic visualization apparently and diagnoses them accurately. The major positive aspect of laparoscopic ultrasound (LUS) is its ability to perform image representation beyond tissue boundaries. Laparoscopic operation was performed using a higher frequency and more close to pelvic organs via laparoscopic access, Characterization of the uterus and lesions is challenged when we want to achieve identification by automatic segmentation for medical applications such that we can locate residual lesions precisely and provide guidance for removal of residual tumor and eliminate its recurrence effectively. A sufficiently accurate segmentation algorithm provides safe and valuable assistance for clinical applications in laparoscopic gynecological surgery.

Laparoscopic ultrasound can provide high-resolution images of the abdominal cavity and contribute to the direct examinations of morphologies and anatomical structures of abdominal viscera and their relative positions [1]. The acquisition of laparoscopic images via ultrasound is very useful for guiding the surgery in preoperative, intraoperative and postoperative procedures [2, 3]. Therefore, the laparoscopic image has been widely applied to many parts of the human body for the diagnosis and treatment of disease on abdominal viscera due to the mini-invasive capability and the ability to obtain high-resolution images. For instance, the laparoscopic image can be used in the disease treatment on gallbladder, such as intraoperative cholangiography, cholecystectomy, choledochal cysts excision and biliary drainage [4, 5]. And it also plays an important role in hepatectomy and hepatic cysts [6] and is significant in splenectomy, spleen fixation, pancreatectomy and pancreatic duct decompression [7]. In addition, it can be also applied in the assignment of the cancer stage, the diagnosis of intraperitoneal tuberculosis, the treatment of peritoneal adhesion, and the resection of various tumors on abdominal organs [8, 9]. Recently, the computer-aided analysis of laparoscopic images has been developed rapidly be-cause the However, laparoscopic surgery presents some inherent limitations such as the lack of tactile feedback in laparoscopic procedures. Surgeons are unable to palpate invisible lesions and can only observe the surface of organ. In addition to the limited surgical field under laparoscopy, the lack of sense of 3-D space is a major problem since two-dimensional display on monitor cannot be seen under laparoscopic visualization or adjacent vessels and tissues injuries in operation [10, 11].Therefore, it is easy to cause misdiagnosis or mistreatment to the lesions, and this motivates the use of an appropriate diagnostic tool in this paper.

There even exists a new intra-operative imaging technique such as the combination of intra-operative ultrasound with laparoscopic surgery and extends tactile sensation. It helps surgeon to detect internal structure of target organ and also support in finding invisible lesions. Furthermore, it compensates defects of laparoscopic technique by two-dimensional cross-section image of ultrasound.

Laparoscopic ultrasound (LUS) was initially used in1984 by Fukuda et al [12]. According to some articles, LUS is regarded as an important technique for laparoscopic surgeries such as liver tumor resection, suspected nodules (metastases) biopsy, and bile ducts detection as expressed in [13–15]. Despite its early introduction, it has been rarely implemented in the past two decades[16]. However, due to recent improvement in skill, equipment and technology, the laparoscopic surgery has been paid more attention by surgeons in clinical application. In addition, it is not only used for intra-operative diagnostic imaging, but also utilized for assisting in minimally invasive of laparoscopic surgery [17–19]. Generally, it has been observed that previous studies of this research field were focused on assessing clinical therapeutic effects such as reducing misdiagnosis, residual or recurrence of myoma with LUS from surgeon’s prospective[10, 11, 19].There are limited literature related to intra-operative ultrasonography based LUS technique including [20].

In [21], laparoscopic ultrasound is utilized to identify patient’s pelvic nodal metastasis with initial period cervical carcinoma. On the basis of LUS identification, abnormal lymph nodes were eradicated laparoscopically for pathological verification by frozen segment. In the case of diagnosis of the nodal metastasis, radical hysterectomy can be terminated. This method is perceptive in discovering macroscopic but not for microscopic metastatic pelvic lymph nodes. The elimination of macroscopic metastatic nodes recognized by laparoscopic technique can be achieved in large proportion of patients. The authors utilized combination of LUS and laparoscopic pelvic lymphadenectomy for supervision of patients with cervical carcinoma [22]. The LUS was applied before performing pelvic lymphadenectomy either laparoscopically or laparotomy. The LUS calculations were evaluated and compared with pathologic results. The results suggest that LUS is extremely responsive in discovering metastatic pelvic lymph nodes. The recognition and elimination of metastatic pelvic lymph nodes laparoscopically permit rapid improvement from the operative process and early instigation of radiotherapy. The comparison of LUS and traditional laparotomy for supervising patients with tubo ovarian abscess (TOA) is explained [23].However, the utilization of TOA includes conventional medical cure with antibiotics, at the present it is believed that surgical intervention should be followed early after the identification. Therefore, open laparoscopy appears to be useful option to conservative laparotomy for management of patients.

LUS is not extensively employed in gynecologic measures, even though the available research recommends that it could strongly be a supportive for determining lymph node metastases for cervical carcinoma, classifying myomas of the uterus if they are not overtly obvious during laparoscopic surgery and evaluating ovarian cysts. The major advantage of LUS is recovering analytical correctness compared to other radiologic methods. Along with that benefit risk factor during surgery is reduced by using LUS. These are the reasons that LUS is best option to use for several clinical applications. However, despite of increasing application laparoscopic ultrasound at abroad, it is less utilized for gynecologic laparoscopic surgery at domestic level. Therefore, in our research we have focused clinical applications that use laparoscopic ultrasound in gynecological laparoscopic surgery.

In this study, we performed different tests for segmentation of the uterus and lesions based on laparoscopic ultrasound images, in order to explore its clinical value in gynecologic laparoscopic surgery, 30 patients for gynecologic laparoscopic surgery were recruited during experiment. Laparoscopic ultrasound examinations were performed in operation and results are summarized in this paper. Our study not only evaluates the intra-operative imaging technique with regards of facilitation of laparoscopic surgeries, but also focuses on depiction of acoustic characteristics of LUS. Along with the results churned from our experimentations, we compared these characteristics with those of trans-abdominal ultrasound (TAS) and trans-vaginal ultrasound (TVUS) to evaluate if LUS can give us more detailed diagnostic information than those of TAS and TVUS do on sonographer’s perspective. The LUS supported in conserving normal tissues as much as possible, preserving its function and lessening surgical trauma.

The remainder of this paper is organized as follows; the second section presents the proposed method, including the detection of the uterus border by the active contour method, refinement of the uterus border, detection of the lumen border with the unsupervised clustering method, and curve smoothing for the two borders; the third section presents the results of the performance evaluation experiments; the fourth section discusses the proposed method in term of its accuracy, robustness, initialization, computational cost, and other issues; the final section gives our conclusions and we suggest possible future improvements to the method.

## Materials and Methods

### 2.1 Procedural Flow Chart and Basic Methodology

**Fig 1:**
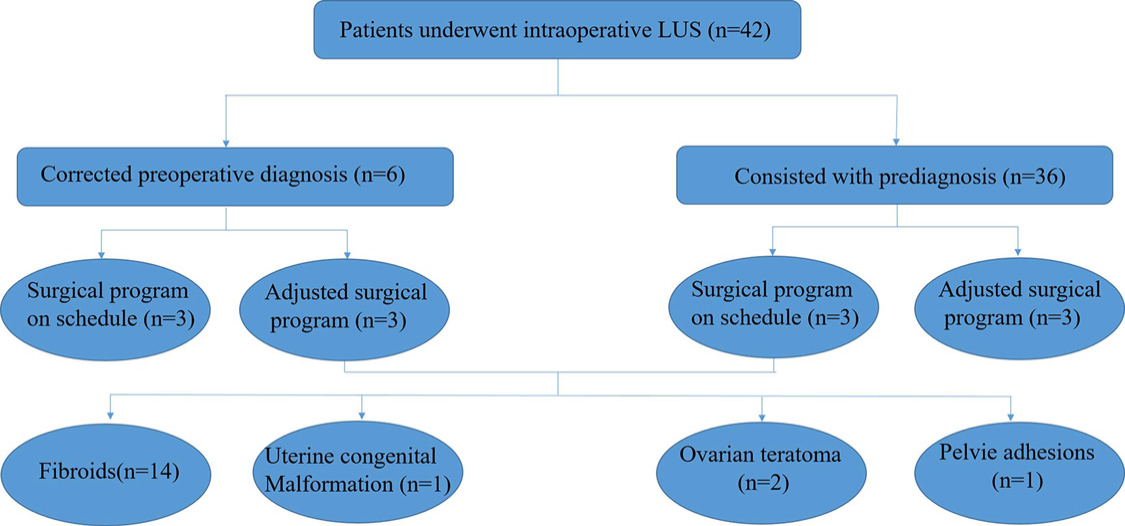
The flowchart of the study. The stages are as follows: laparoscopic ultrasound images provide the raw image of the uterus; detection of uterus border via an active contour model; outputting the outline of the detected uterus for laparoscopic diagnosis and surgical guidance and analysis (this may be used for detecting lesions on the uterus or determining the abnormality of the uterus).

In this section, we introduce an method to segment uterus in laparoscopic images. Fig 1 illustrates the flowchart of the proposed method. Firstly, the specular reflection components on the surface of organs and tissues in the gray laparoscopic image are removed for reduce the influence of position variance of the light source and the camera in the surgery. Then a thresholding algorithm is applied to divide the image into two classes for the purpose of finding the region of interest (ROI) containing uterus. Next, three kinds of features from uterus are extracted and then applied in the support vector machine in order to discriminate between the uterine region and non-uterine region. Finally, the snake algorithm is developed for detecting the edge of uterus.

In gynecologic laparoscopy, uterus segmentation plays a vital role in the computer-aided analysis of laparoscopic images, because uterus is an anatomical marker used to locate the surrounding organs and tissues, such as ovarium, uterine tube, bladder, etc. And it also administer to the discrimination of the uterine tumor. Moreover, it has the particular importance in 3D reconstruction required for augmented reality in laparoscopic surgery [24]. In this paper, we developed a method to segment the uterus in laparoscopic images based on the active contour algorithm and the SVM classifier using three kinds of image features. The performance evaluation of our method is tested over 100 images from 30 patients, and the results are acquired from the comparison between two methods: our method and the manual drawing method by one clinical doctor. This study was approved by The First affiliated hospital of Guangzhou University, Guangzhou, and written informed consent was obtained from each subject before attending this study. In the experiments, linear regression and Bland-Altman analysis are applied to respectively investigate the correlation and the agreement between two methods. In addition, the similarity of uterus segmented by the two methods are evaluated separately by Dice index and Jaccard index.

### 2.2 Laparoscopic Ultrasound (LUS)

At the beginning of laparoscopic surgery or after the initial surgical procedure, laparoscopic ultrasound examinations were performed by two medical experts. Laparoscopic ultrasound probe was sterilized by epoxyhexane for the preparation of experiment. To complete experiment ultrasound device is operated by both gynecologist and sonographer. The gynecologist operated laparoscopic ultrasound probe on operating table and a sonographer coordinated with him closely for successful operation.

In the LUS examinations, laparoscopic ultrasound probe was inserted via a trocar (10.0mm in diameter) and placed on pelvic organ surface to scan under direct laparoscopic visualization. The probe head could bend to the different axial both horizontally and vertically for multi-directions, multi-angles scanning in the pelvic cavity by manipulating the adjusting device on the probe handle. This is because the probe can be adjustable to 90°in four directions in order to allow maximum tissue contact. The instruments were adjusted in such a way so that pelvic organs and structures can be displayed on the monitor clearly.

The Esaote Bio-sound MyLab 30CV portable ultrasound machine, abdominal transducer with multi-frequencies (3.5–6 MHz), atrans-vaginal transducer with multi-frequencies (5–7 MHz), a linear-arraylaparoscopic ultrasound transducer, dedicated model Esaote S.P.A, LP323 REP 9600162000 with multi-frequencies(7.5-10MHz) with range of 10.0mm in diameter.

### 2.3 Active contour model for uterus segmentation

In order to improve the efficiency of the diagnosis in laparoscopic ultrasound examinations, computer-aided approaches are introduced to compute the size, number, shape, location and internal acoustic characteristics of the lesions were observed by two-dimensional gray-scale ultrasound.

The gynecological region of interest (ROI) may be composed of disjunct regions, and one of them is the uterus region. Therefore we should find each separated subregion of ROI, and determine which subregion best represents the uterus. The border of uterus is determined by a coarse-to-fine strategy. A doctor draws points on the border of the uterus based on their specialized knowledge in order to determine the coarse location of the uterus border. And a convex polygon can be constructed by considering these points as the apexes. Then the boundary of the convex polygon can be considered as the initial front, which can be evolved by the active contour method–the snake algorithm [24]. In the proposed snake algorithm, the energy function of the contour can be divided into two parts: the internal energy E_1_ and the image energy E_2_, formulated as

$$E=\;\int_{0}^{1}\;E_{1}\left(x\right)ds+\alpha \;\int_{0}^{1}\;E_{2}\left(x\right)ds,$$ (1)

where *α* is the weight parameter set at 1.7, *x* is the coordinate vector, and *s* is the curve parameter. The internal Energy E_1_ can describe the tension and the stiffness of the front, formulated as

$$E_{1}\left(x\right)=\frac{dx\left(s\right)}{ds}+\;\beta \frac{d^{2}x\left(s\right)}{ds^{2}},$$ (2)

where *β* is the weight parameter set at 0.7.

The image energy can describe the characteristics of image intensity and gradient around the border of lesion, formulated as

$$E_{2}\left(x\right)={\left(\frac{G(x)}{\left|G\left(x\right)\right|}\right)}^{2}+\;{\left(\frac{I(x)}{\left|I(x)\right|}\right)}^{2},$$ (3)

where G(x) and I(x) are the gradient value and pixel value at the coordinate x in the ultrasound image, respectively.

The border of uterus can be considered as the front when the snake algorithm is converged, which indicate the segmentation region of the uterus.

### 2.4 Experiments

This study was approved by The First affiliated hospital of Shenzhen University, Shenzhen. All the laparoscopic ultrasound images data were collected over a commercially available machine (Rudolf Corp., Rancho Cordova Germany). The laparoscopic images were then saved as AVI format into CDs for off-line analysis. All the codes in this work were implemented by Matlab 2013 on a desktop computer with Pentium(R) Dual-Core CPU (3 GHz) and 2 GB memory. All the experiments were tested on 100 in-vivo images acquired from 42 patients (age 36.8 ± 5.6 years old).

**Fig 2:**
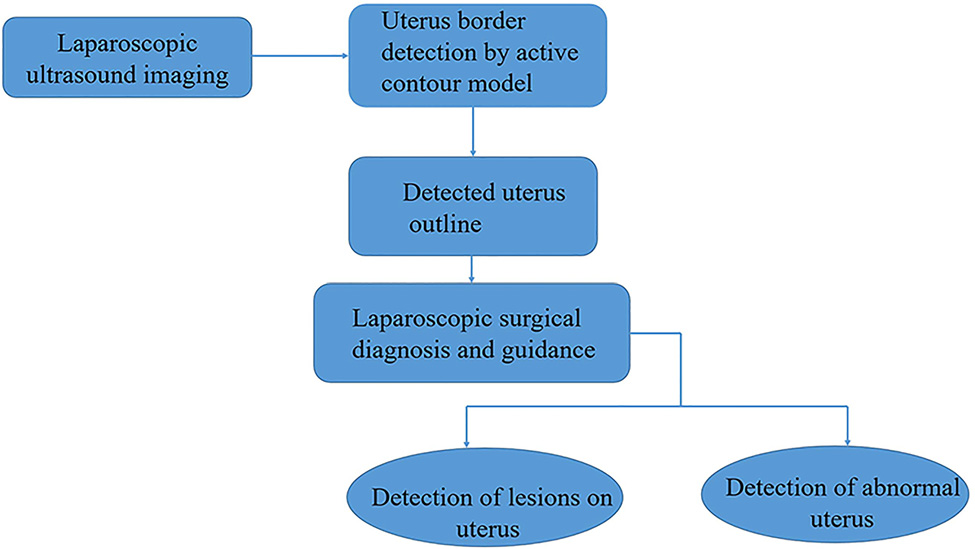
Classification of intra-operative laparoscopic ultrasound examination of 42 women subjects. The gynecological cases included: uterine fibroids (25 cases), congenital uterine malformations (4 cases), ovarian endometriomas (4 cases), pelvic adhesions masses (2 cases), ruptured ectopic pregnancies (2 cases), and small nodules biopsy on pelvic wall (1 case).

In our experiment, 42 subject cases were analyzed under gynecologic laparoscopic surgery; intra-operative laparoscopic ultrasound examination was performed at our hospital from August 2011 to August 2013. The classification of intra-operative laparoscopic ultrasound examination of 30 women subjects is shown in Fig 2. The average age of the patients was 39 years old (range between 23 and 51). including: (a) 25 cases of uterine fibroids, all were multiple fibroids, diameter 6-65mm; (b) 4 cases of congenital uterine malformations, out of them 3 were incomplete mediastinal uterus and 1 was complete septate uterus, (c) 2 cases were bilateral (diameter 19-43mm), (d) 4 cases of ovarian endometriomas, (e) 2 cases of pelvic adhesions masses, (f) 2 cases of ruptured ectopic pregnancies; (g) 1 case of small nodules biopsy on pelvic wall. Routine preoperative abdominal or trans-vaginal ultrasound was performed, and postoperative pathologic results confirmed in all cases (Fig 2).

There are 4 cases of uterine congenital malformations diagnosed by preoperative ultrasound and scheduled for laparoscopically assisted vaginal hysteroscopic septum resection. Of these, 3 cases were of incomplete mediastinal uterus and dissected by LUS, and 1 case was of complete septate uterus. While one case with uneven thickness of mediastinum found by LUS examination, the upper segment of septum (adjacent to uterine fundus) was thicker than 18 mm, the lower segment (corpus segment) was thin like an “inverted triangle”. It is considered that only the lower part of the septum was resected in implementation of hysteroscopic septum resection. For our experiment on active contour segmentation of uterus, we decided to choose the 4 cases of congenital uterine malformations and 2 cases of pelvic adhesions masses, making a total of 6 cases altogether. The two types of cases were classified into Group A and Group B respectively.

## Results

**Fig 3:**
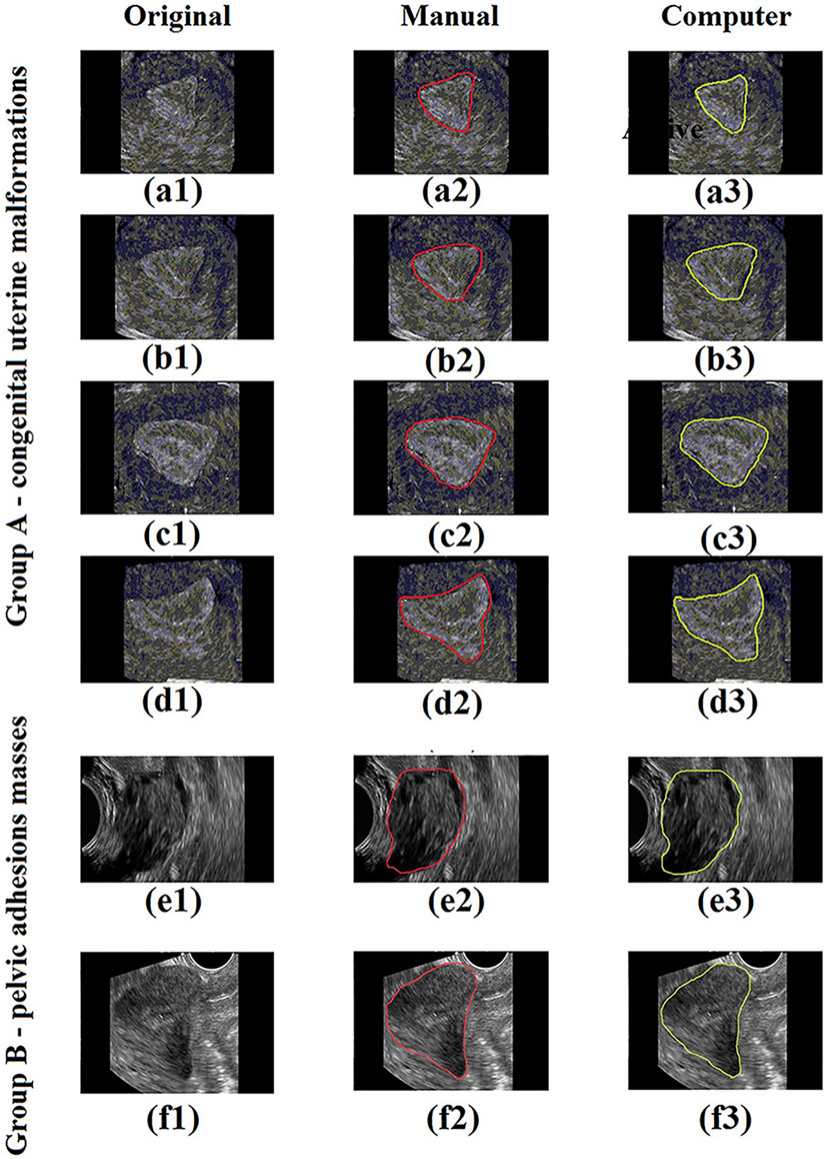
Gynecology ultrasound scans with segmentation of uterus by active contour model. (a1), (b1), (c1), (d1), (e1) and (f1) in the first column -are original images. (a2), (b2), (c2), (d2), (e2) and (f2) in the second column are images with uterus edge drawn by the doctor. (a3), (b3), (c3), (d3), (e3) and (f3) are images with uterus edge detected by our method. (a) to (d) represents Group A, and (e) to (f) represents Group B of our experiment.

Based on the 6 human subjects with gynecological conditions, Fig 3 shows some results of the uterus segmentation for each of these cases. We quantified four measurements to evaluate the performance of the proposed method: uterine area (UA), uterine maximum diameter (UMXD), uterine minimum diameter (UMID) and angle of the uterine long axis (AULA). UA is the area within the uterine. UMXD/UMID is the maximum/minimum diameter of the uterus border. AULA is the angle of the major axis of the ellipse which is fitted based on the least square criterion [25] by the uterus border. The above measurements acquired from two methods are compared: our method and the manual drawing method by one clinic doctor. And the latter is considered as the ground truth in our paper. Then the comparative results are analyzed by two evaluation approaches: the linear regression and the Bland-Altman analysis [26]. In the linear regression, we investigate the correlation coefficients *r* because this index represents the correlation between the two methods. For UA, UMXD, UMID and AULA shown in Table 1, values of *r* ranges from 0.80 to 0.93. In the Bland-Altman analysis, the agreement between the two methods can be analyzed by the 95% confidence interval CI and the percentage of points r_95%_ in CI. For the four measurements, the values of r_95%_ ranges from 81.34% to 94.56% considering all of the case studies, and the values of root mean square error (RMSE) ranges from 8.34 to 10.72 for measurement UA, 0.46 to 0.71 for measurement of UMXD, 0.12 to 0.24 for measurement of UMID, and 7.89 to 10.3 for measurement of AULA, respectively. In addition, we use the Dice index [27]and the Jaccard index [28] to analyze the similarity of uterus segmented by the two methods. In Table 2, the mean value and standard variation of the Dice index are 89.58% and 3.13% respectively, and those of the Jaccard index are 88.57% and 3.98% respectively.

**Table 1:** The results of the linear regression and the Bland-Altman analysis.

No.	Measurement	Linear Regression			Bland-Altman	
		Linear Function	*r*	RMSE	*r*_95%_	CI
1	UA	*y* = 0.82*x*+1.19	0.85	10.72	92.04%	[-1.23,2.48]
	UMXD	*y* = 0.93*x* -0.36	0.88	0.46	82.45%	[-0.34,0.45]
	UMID	*y* = 0.85*x* -4.62	0.91	0.12	94.56%	[-2.45,1.46]
	AULA	*y* = 0.87*x* +8.23	0.89	8.61	92.04%	[-2.75,0.45]
2	UA	*y* = 0.78*x* -13.61	0.81	9.83	87.12%	[-1.23,1.36]
	UMXD	*y* = 0.83*x* +3.63	0.82	0.70	85.27%	[-0.45,1.86]
	UMID	*y* = 0.75*x* +19.26	0.90	0.21	86.12%	[-1.64,2.39]
	AULA	*y* = 0.91*x* +10.41	0.85	9.32	91.03%	[-0.45,1.06]
3	UA	*y* = 0.96*x* -0.35	0.87	10.2	89.34%	[-0.87,0.84]
	UMXD	*y* = 0.83*x* 1.93	0.80	0.71	81.34%	[-1.38,1.58]
	UMID	*y* = 0.81*x* -4.67	0.91	0.16	90.34%	[-0.98,2.49]
	AULA	*y* = 0.79*x* +5.62	0.93	9.31	86.45%	[-2.34,0.47]
4	UA	*y* = 0.70*x* -1.68	0.84	9.45	83.00%	[-2.85,3.14]
	UMXD	*y* = 0.85*x* +18.54	0.81	0.68	90.45%	[-0.56,2.45]
	UMID	*y* = 0.75*x* +7.23	0.86	0.24	82.75%	[-1.55,0.51]
	AULA	*y* = 0.91*x* +0.47	0.90	10.3	85.83%	[-0.57,2.48]
5	UA	*y* = 0.86*x* -3.46	0.82	8.34	90.34%	[-0.87,3.92]
	UMXD	*y* = 0.79*x* +9.54	0.93	0.69	87.23%	[-0.91,3.18]
	UMID	*y* = 0.97*x* +0.14	0.81	0.15	86.24%	[-1.06,2.35]
	AULA	*y* = 0.92*x* -9.45	0.92	7.89	90.07%	[-2.56,0.34]
6	UA	*y* = 0.77*x* +1.23	0.84	9.32	87.23%	[-0.91,1.46]
	UMXD	*y* = 0.79*x* +9.45	0.82	0.62	88.56%	[-1.38,3.10]
	UMID	*y* = 0.82*x* -6.45	0.81	0.13	90.23%	[-2.37,2.00]
	AULA	*y* = 0.85*x* +3.55	0.93	8.01	87.12%	[-1.57,1.39]

**Table 2:** The results of the Dice index and Jaccard Index.

No.	Dice Index	Jaccard Index
1	90.23% ± 3.42%	87.13% ± 2.49%
2	88.12% ± 4.21%	89.21% ± 4.31%
3	90.92% ± 1.35%	89.31% ± 5.34%
4	88.67% ± 2.32%	88.21% ± 4.93%
5	91.31% ± 2.57%	90.21% ± 2.36%
6	88.23% ± 4.93%	87.35% ± 4.42%
Maximum deviation	9.97%	7.93%
Mean	89.58% ± 3.13%	88.57% ± 3.98%

**Fig 4:**
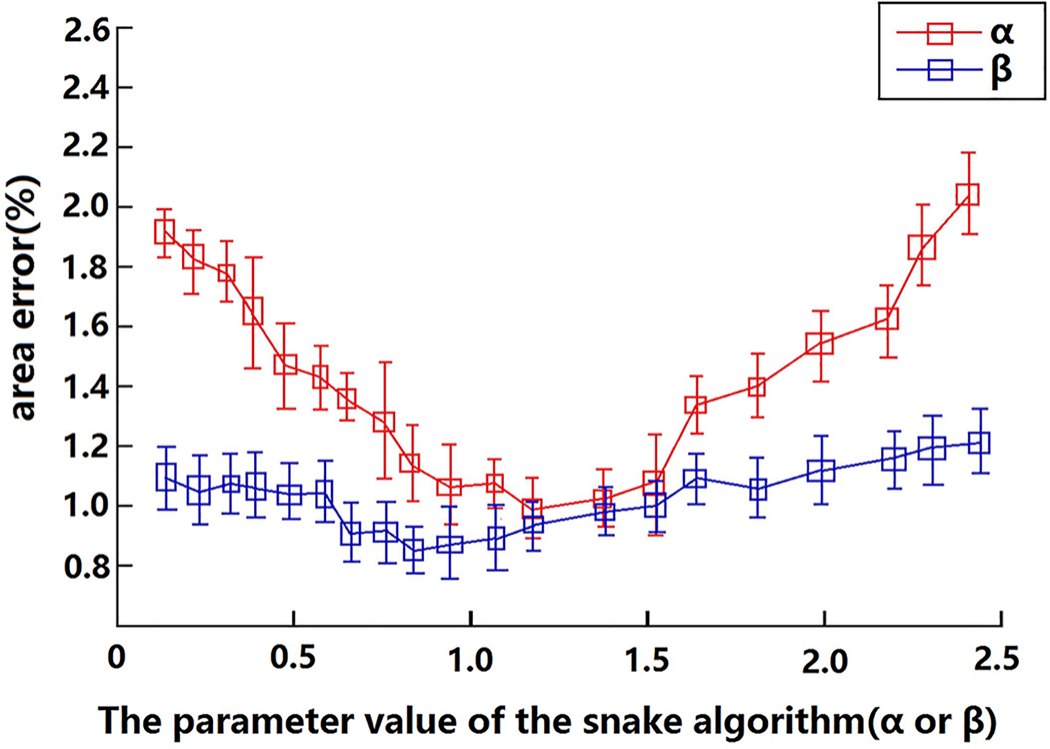
The error variation in different values of *α* and *β*. The error is represented by the percentage that the area difference occupies in the image, where the area difference is the difference between areas computed by our method and drawn manually.

In addition, in order to choose the proper values of the parameters α in Eq (1) and β in Eq (2), we evaluated the influence from the variation of the two parameters on the performance of our method. We investigate the error variation in different values of α, where error is represented by the percentage that the area difference occupies in the image and area difference is the difference between areas separately computed by our method and drawn by manual. As seen in Fig 4, we can have the smaller values of error when *α* is close to 1.23. Thus we set *α* at 1.23 in our method. Similarly, we also study the error variation in different values of *β*. From the same Figure, we can have the smaller values of error when *β* is close to 0.91. Therefore, we set *β* at 0.91 in our method.

The 2 cases of extensive pelvic adhesions with large “inflammatory mass” (diameter>100mm) were observed under laparoscopic lysis surgery guided by LUS. It showed that the complex pelvic masses containing both solid and cystic components were adherent to pelvic organs and wall, which were distinguished from the normal pelvic anatomic structures by preoperative US. Additionally, the 2 cases of ruptured tubal pregnancy were detected and their sizes were about 55×30mm, 50×35mm. The hematoma-like masses with blurred boundaries were found under laparoscopic visualization. We performed the following operations: i) The swelling fallopian tubes that were mixed in the masses can be identified by LUS scanning; ii) we positioned the rupture of fallopian tubes; and facilitate their removal with LUS guidance.

The checking of initial inflammatory effusion suggested that there was still 1 patient of pelvic nodules biopsy having larger cystic lesion. The preoperative diagnosis was corrected subsequently. Whereas, with the real-time LUS guidance, adhesions were lysed step by step, all effusion (thick, chocolate-like fluid) was aspirated and lesions were removed completely. In 1 case of laparoscopic ovarian tumor resection, several small nodules were found on the pelvic side wall adjacent to the ovarian mass and lymph node metastases were in suspicion. Intra-operative LUS examination was applied in assessment of suspicious nodules guiding to check the nodules in biopsy and inflammatory enlarged lymph nodes were confirmed subsequently.

## Discussion

This discussion section is divided into following two sections: a) evaluating the performance of the computational segmentation approach; and b) implementation of the technique and how it assists in gynecological diagnosis.

### 4.1 Accuracy and robustness of active contour segmentation of uterus

In this section, we discuss the accuracy and robustness of our method. Our method’s accuracy is evaluated by comparing the performance of our method and the manual segmentation by one clinical doctor (ground truth), and the results from 100 laparoscopic images from 5 patients are investigated by two evaluation approaches (linear regression and Bland-Altman Analysis). In the linear analysis, the correlation coefficients are larger than 0.82 for the four measurements (UA, UMXD, UMID and AULA), representing that our method is well correlated with the manual method. And in the Bland-Altman analysis, most points (>84%) fall in the 95% confidence interval, and it means that there is a good agreement between our method and manual method. In addition, Dice index and Jaccard Index can be used to describe the shape similarity between two uterus in the image, where one is segmented by our method, and other is traced by the doctor. The results show that the Dice index and Jaccard Index are 88.06% and 86.32%, respectively. Therefore, our method has similar performance comparing to the manual segmentation method by the clinical doctor.

The features of uterus by (UA, UMXD, UMID and AULA) facilitate us to improve the performance of our method to discriminate between the uterus and other organs, tissues and lesions. In addition, in the border detection of uterus, the snake algorithm is used to make the initial contour approximate to the uterus border by the iterative process. It can adjust the contour evolution by the internal energy function to find the weak uterus border, which is caused by its relative position with other visual-similar tissues, the intervention by lesions and the luminance in-sufficiency resulting from the improper position of the camera in vivo. Furthermore, we investigate the between-patient difference of our method’s performance. Table 1 showed that the differences for the agreement and correlation between patients are smaller than 5% and 4.00 respectively. And in Table 2, the maximum deviation of the Dice index and Jaccard index computed from different patients are 9.97% and 7.93%, respectively. So we can conclude that our method has approximately similar performance in the images from different patients.

### 4.2 Use of laparoscopic ultrasound image processing in diagnosing gynecologic conditions

Laparoscopic ultrasonic probe has a higher frequency. In addition, it can be inserted into the pelvic cavity via trocar and to be placed directly on the surface of pelvic organs and for scanning. Owing to the non-interference of the abdominal wall and bowel gas, the border and internal acoustic characteristics of the uterine or ovarian tumors can be displayed by LUS much better than by TAS (transabdomialsonography) or TVUS (transvaginal ultrasound). Therefore, it is helpful in assessing the characteristics of tumor (benign or malignant) and its location. Our study showed that intra-operative LUS has not only helped in the diagnosis of pelvic lesions according to its sonographic appearances, but it was also useful in detecting more small residual lesions and determining the margin of surgical resection accurately under the premise of ensuring complete removal of the tumor and reducing the recurrence. It can assist in preserving normal tissues as much as possible, maintaining their physiological functions, and reducing surgical trauma [29].

#### 4.2.1 Group A: Diagnosis of Congenital Malformations

It was observed that when the probe was placed on the surface of the uterine fundus, the LUS could display the fundus, corpus and cavity of the uterus more clearly than TAS and TVUS. Therefore, it could be used to correct uterine congenital malformations in laparoscopic assisted vaginal hysteroscopy. The four cases for laparoscopic assisted vaginal hysteroscopic septum resection were scheduled in order to minimize the risk of uterine perforation. The LUS examinations were performed and intrauterine surgical procedures were monitored by real-time LUS. When the LUS probe was inserted into the pelvic cavity via corcar and placed on the surface of uterine fundus, the septum could be displayed extending from the fundus to cervix, and the uterine cavity was divided into two parts. The thickness and length of septum could be measured accurately. The 3 cases of septate uterus with uniform thickness of mediastinum and mediastinal excision were taken completely by hysteroscopy with the guidance of LUS.

However, one case of uneven thickness of mediastinum was also observed. The thickest part of mediastinum (uterine fundus segment) was more than 18mm wide in comparison with the thinner uterine corpus segment as per display mentioned by LUS look like an “inverted triangle”. Taking into account of avoiding of uterine perforation, only the lower part of the corpus segment of septum was resected for treatment [30]. The ovary varies (morphological change occurs) during a menstrual cycle. Owing to the coverage of ovarian capsule, it is difficult to distinguish whether the ovarian mass was acting as physiological or pathological feature. The internal structure of ovary and small mass in the ovary were also displayed lucidly by LUS than preoperative TAS and TVUS.

#### 4.2.2 Group B: Analysis of Pelvic Adhesions Masses

Two patients of complex pelvic adhesions were observed having multiple pelvic organs with encapsulated effusion forming caused by the history of chronic inflammation, surgery or endometriosis. It was difficult to identify the anatomical relationship even directly under laparoscopic visualization. Laparoscopic lysis surgery was implemented in those two patients. Surgeons need to know about the local surgical anatomy, the vascular distribution, the tumor’s location and adjacent is in real time, because the blunt dissection of severe adhesion might damage the vital pelvic vessels and structure. Therefore, LUS was used in guiding the laparoscopic lysis surgery.

Once the initial separation of adhesions and aspiration of effusion in one patient were obtained, LUS can be used to discover that there were still presences of larger endometrial lesion. LUS enhanced the surgeon’s confidence and helped them to recognize the pelvic structure and avoid vital vessels. In subsequent operation, It helped us continuously to find the appropriate path to separate the adhesions (cutting and burning) layer by layer, and also at the same time eliminate the residual lesions and avoid complications [31]. In one case of ovarian cystadenoma patients after laparoscopic resection, several small raised nodules to be found on pelvic wall near the ovarian masses having suspected metastases. The intra-operative LUS was applied for assisting in differential diagnosis of small lesions on pelvic wall. It showed that the nodules located under plasma membrane were hypoechoic with envelope and neat border. Moreover, following LUS guided biopsy, it was confirmed by pathologic results [32].

### 4.3 Improvements for intra-operative LUS examination

Evidently, LUS could also be used in guiding laparoscopic surgery in real-time by defining the border of surgical resection precisely for assisting in surgical resection or biopsy. The LUS not only helped to correct preoperative diagnosis and to reduce misdiagnosis rate, but also eliminate lesions and diminish recurrence. Additionally, to minimize surgical trauma, to preserve organs function, reflect the less invasive criteria of laparoscopic surgery.

The intra-operative LUS examination that was discussed above has many advantages. But during experiment of LUS examination, some problems were also observed which need to be resolved. The LUS linear-array scanning transducer was thin rod-shaped with a narrow head not easy to contact with organs surface closely. The size and number of ultrasonic transmission chips at the probe head were tiny and fewer in numbers, so the scope of sonographic deployment is limited. Interpretation of the sonography was still needed to be in adaptation. Due to these issues, we fixed LUS transducer on one hand and pushed the organ or lesions closely to the scanning plane of the probe head as possible on the other hand to scan the interested region for our experiment. In addition, saline injection into the space of pelvis was also taken for help in further lesion deployment. Overall, LUS examination is not standardized for reference yet and has very rare deployment in recent time.

## Conclusion

The uterus segmentation in gynecologic laparoscopy is of importance to quantitatively analyze the nearby organs, tissues and lesions. In this paper, we develop a method for the segmentation of uterus by the active contour technique based on the Knass snake algorithm. Then the proposed method is compared with the manual drawing method by one clinical doctor in order to evaluate our method’s performance. The results analyzed by linear regression, Bland-Altman analysis, Dice index and Jaccard index show that the accuracy and robustness of our method are at a high level.

Our research has formulated a new approach of guiding laparoscopic surgery and gynecologic diagnosis. This study utilizes 42 women patients, among these subjects 6 cases corrected preoperative analyzes after LUS examination and 18 cases regulated actual surgical program consequently. In our conducted experiment, during laparoscopic operation when frequency is high or near to pelvic organs by accessing through laparoscopic, LUS assists gynecologists to determine tiny residual lesions using laparoscopic visualization clearly and analyzes them correctly by first locating the borders of the uterus accurately. Furthermore, LUS is also assisting to find residual lesions specifically and offer assistance to eradicate residual tumor and remove its reappearance effectually.

The LUS has provided us a better intra-operative assessment of definite diagnosis, precise guidance in laparoscopic surgery and reduction of risk for laparoscopic ultrasound in gynecological laparoscopic surgery. During performing experiment, it was observed that LUS offers more protected and important support for clinical applications in laparoscopic gynecological surgery than trans-abdominal ultrasound (TAS) and trans-vaginal ultrasound (TVUS). In future, laparoscopic ultrasound will be based on high-quality vision technology in the field of “minimally invasive" in gynecologic surgery.
